# shinyBN: an online application for interactive Bayesian network inference and visualization

**DOI:** 10.1186/s12859-019-3309-0

**Published:** 2019-12-16

**Authors:** Jiajin Chen, Ruyang Zhang, Xuesi Dong, Lijuan Lin, Ying Zhu, Jieyu He, David C. Christiani, Yongyue Wei, Feng Chen

**Affiliations:** 1 Department of Biostatistics, School of Public Health, State Key Laboratory of Reproductive Medicine, Nanjing Medical University, Nanjing, China; 2 Department of Epidemiology and Biostatistics, School of Public Health, Southeast University, Nanjing, China; 3 Department of Environmental Health, Harvard School of Public Health, Boston, MA USA; 4 Jiangsu Key Lab of Cancer Biomarkers, Prevention and Treatment, Jiangsu Collaborative Innovation Center for Cancer Personalized Medicine, Nanjing Medical University, Nanjing, China

**Keywords:** Bayesian network, Visualization, Inference, Online tool, R package

## Abstract

**Background:**

High-throughput technologies have brought tremendous changes to biological domains, and the resulting high-dimensional data has also posed enormous challenges to computational science. A Bayesian network is a probabilistic graphical model represented by a directed acyclic graph, which provides concise semantics to describe the relationship between entities and has an independence assumption that is suitable for sparse omics data. Bayesian networks have been broadly used in biomedical research fields, including disease risk assessment and prognostic prediction. However, the inference and visualization of Bayesian networks are unfriendly to the users lacking programming skills.

**Results:**

We developed an R/Shiny application, *shinyBN*, which is an online graphical user interface to facilitate the inference and visualization of Bayesian networks. *shinyBN* supports multiple types of input and provides flexible settings for network rendering and inference. For output, users can download network plots, prediction results and external validation results in publication-ready high-resolution figures.

**Conclusion:**

Our user-friendly application (*shinyBN*) provides users with an easy method for Bayesian network modeling, inference and visualization via mouse clicks. *shinyBN* can be used in the R environment or online and is compatible with three major operating systems, including Windows, Linux and Mac OS. *shinyBN* is deployed at https://jiajin.shinyapps.io/shinyBN/. Source codes and the manual are freely available at https://github.com/JiajinChen/shinyBN.

## Background

Bayesian networks have become one of the most commonly used models for the modeling and reasoning of uncertain systems. In the biomedical field, Bayesian networks are successfully applied to assess the risk of disease and explore the relationship between genotypes and phenotypes [1, 2]. However, the inference and visualization of Bayesian networks is not user friendly. *SMILE* (Structural Modeling, Inference, and Learning Engine) is a causal discovery engine [3] and is easily embedded into the other tools, such as *jSMILE*, a Java implementation of *SMILE*, and *rSMILE*, an R package connecting to *jSMILE* [4]. However, since *SMILE* has been shifted from open license to commercial version (product brand: *GeNIe*), *rSMILE* and *jSMILE* are no longer maintained. *BayesianNetwork*, an R/Shiny web widget to construct Bayesian network [5], while the connections between nodes are nondirectional, and only one predictor variable can be considered for outcome inference which hinders its application in real-word medical studies. In addition, there are some commercial products for Bayesian network analysis which require complex installation (Table 1). To solve these inconveniences, we developed *shinyBN*, an online tool based on R and Shiny for interactive inference and visualization of Bayesian network, incorporating multiple types of inputs, flexible parameter settings, and multiple combinations of outcomes.

**Table 1:** Existing tools for Bayesian network analysis

Tool	Latest version	Free	Open source	Web based	R API	Interactive visualization	Batch inference
jSMILE/ rSMILE	2009^a^	Y	N	N	Y	Y	Y
Netica	2018	N^b^	N	N	N	Y	N
BayesiaLab	2018	N	N	N	N	Y	Y
BayesianNetwork	2018	Y	Y	Y	Y	N	N
GeNIe/ SMILE	2019	N^c^	N	N	Y	Y	N
Bayes Server	2019	N	N	N	Y	Y	Y
shinyBN	2019	Y	Y	Y	Y	Y	Y

^a^Stop maintenance

^b^Maximal 15 nodes allowed for free version

^c^Free only for academic community

## Implementation

### Overview of *shinyBN*

**Fig. 1:**
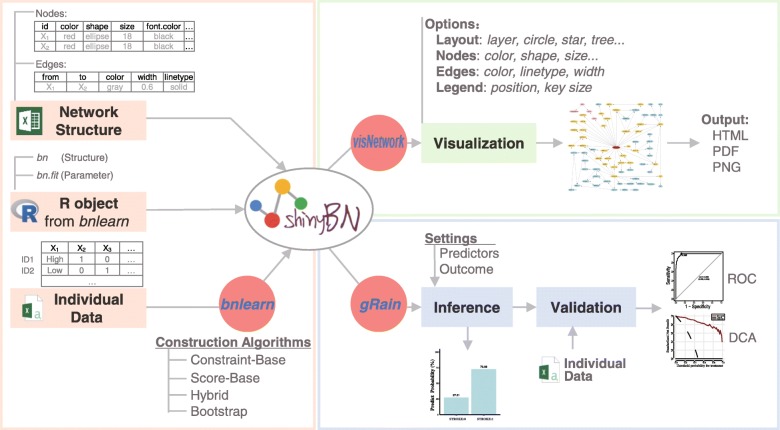
The flow chart of the proposed *shinyBN* application

*shinyBN* was developed with five R packages:

- *bnlearn* for structure learning and parameter training [6];
- *gRain* for network inference [7];
- *visNetwork* for network visualization [8];
- *pROC* for plotting receiver operating characteristic (ROC) curves [9];
- *rmda* for plotting the decision curve analysis (DCA);

and was further wrapped by R/Shiny, a framework to build interactive web applications by R [10]. By using these packages, *shinyBN* could construct the Bayesian network by the uploaded structural information from Excel file or R object, learning the Bayesian network by individual data, visualize and customize the network illustration, and implement the network for outcome inference. A flow chart of the proposed *shinyBN* is shown in Fig. 1. *shinyBN* is compatible with three major operating systems and popular browsers (Additional file 2).

### Network input

*shinyBN* supports three types of input:

- Microsoft Excel file, which has network structural information and properties of the nodes (size, color, shape) and edges (color, width and line type);
- *bnlearn* output object that embeds Bayesian network (class *bn* or *bn.fit*);
- csv file with individual data for Bayesian network structure learning and parameter training. The data is an N × M matrix with discrete data, where N is the number of observables and M is the number of the features (nodes).

### Network construction

Bayesian network constructions are performed using the methods in the *bnlearn* R package [6]. Users can select constraint-based algorithms, score-based algorithms or hybrid algorithms to train the network structure and incorporate structural priors by setting whitelists (included in the graph) and blacklists (excluded from the graph), and the bootstrap approach is supported in *shinyBN* as well [11]. Parameter estimation via either maximum likelihood estimation or the Bayesian method is supported in *shinyBN*. The structure information (nodes, edges) of constructed network can be further extracted for visualization using *visNetwork*. The network can be directly transformed to class *grain* that met the requirement of the input for *gRain* package and perform inference.

### Network visualization

Network visualizations are based on the *visNetwork* R package using *vis.js* JavaScript library [8]. Once the input is uploaded to the server, a visualization of the network with default settings is automatically rendered. The properties of the nodes and edges can be modified by changing the corresponding settings. Node color can be defined individually, by color palettes that meet scientific journal requirements, or by the dominant colors automatically extracted from the uploaded picture. The widths of the connections can be defined manually or corresponding to the strength of the probabilistic relationships. For a better presentation, graph layouts can be modified by the default layouts or, conveniently, by mouse drag and drop. A high-resolution network graph can be downloaded from *shinyBN*.

### Outcome inference

Inferences are performed using the junction tree algorithm in *gRain* R package [7]. It transforms a Bayesian network model into a tree, combines the efficiency of belief propagation and the sum-product method to allow the efficient computation of posterior probabilities.

By selecting the nodes of interest as outcomes, defining the factors (nodes) as predictive variables, setting the values accordingly as evidence, the predicted results will be displayed in a bar plot or a probabilistic table. Marginal and joint prediction results for multiple outcomes can be output. In addition, *shinyBN* supports external validation sets uploaded for batch inference and outputs the inference results, an ROC curve, a DCA curve and other evaluation indices. Publication-ready high-resolution figures can be downloaded from *shinyBN*.

### Timing evaluation

The performance of the application largely depends on the configurations of the computer. In order to improve the performance, the shiny server is upgraded with 8GB of RAM. We evaluated the timing of *shinyBN* by using several publicly accessible networks with different number of nodes (Table 2).

**Table 2:** Timing evaluation of *shinyBN*

Network	Number of nodes	Number of edges	Time for visualization	Time for single inference
Cancer	5	4	1 s	2 s
Child	20	25	1 s	2 s
Hailfinder	56	66	1 s	2 s
Pathfinder	109	195	1 s	2 s
Diabetes	413	602	2 s	7 s
Munin (full network)	1041	1397	2 s	12 s

## Results

### Real data application

Stroke is a severe complication of sickle cell anemia (SCA) that can cause permanent brain damage and even death. By integrating 108 SNPs from 39 candidate genes and clinical characteristics from 1398 individuals with SCA, Sebastiani et al. constructed a Bayesian network to predict the risk of stroke, which achieved an excellent accuracy of 98.2% [1].

**Fig. 2:**
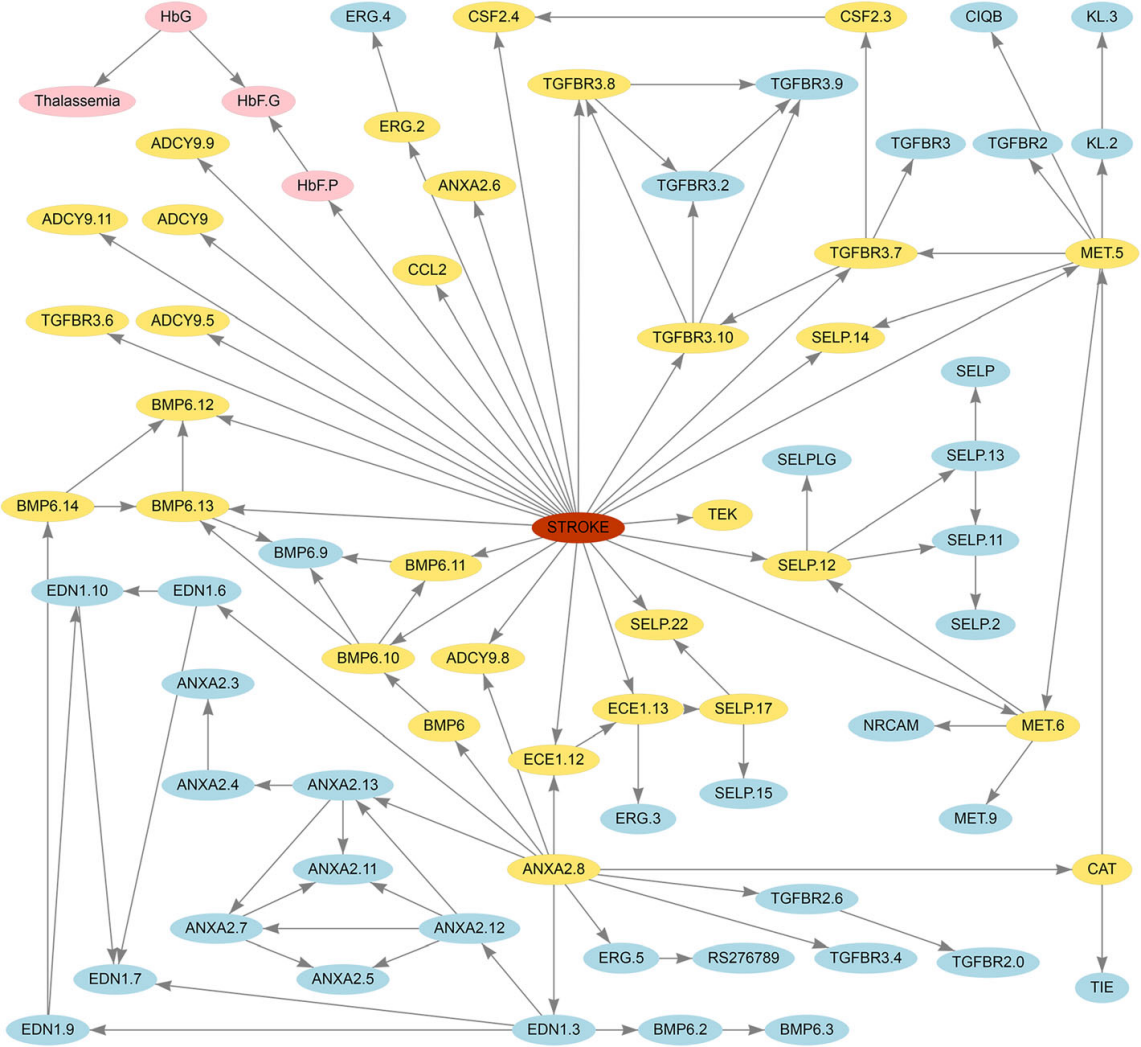
The stroke network rendered by *shinyBN*

First, the network model was replicated using the information from the original study and uploaded to *shinyBN*. Size and color of nodes and width of edges could be modified for a better presentation. In the example, the color of the nodes for the clinical characteristics was set to pink, and the color of the Markov blanket for stroke, which directly associated with stroke, was set to yellow. The layout of network was manually adjusted by the mouse drag and drop (Fig. 2). By setting the evidence for some candidate gene loci, the predicted probabilities for stroke are displayed in a table and a bar plot (Additional file 3). The network can be downloaded as an HTML file for high-resolution figures or in as an Excel file for network structures. Furthermore, we simulated random data from the stroke Bayesian network with a missing rate of 40% for each variable and then uploaded it to the server as a pseudo external validation set. The ROC plot (Additional file 4) and the DCA plot (Additional file 5) were displayed, and the batch inference results were download as a comma-separated values file.

## Conclusions

In conclusion, we developed an online application, *shinyBN*, to construct and illustrate a Bayesian network with high scalability. *shinyBN* supports multiple types of input and provides flexible settings for network rendering and inference. A real data application confirms that the Bayesian network can be used for omics data modeling. By integrating several packages, *shinyBN* is a practical pipeline for Bayesian network modeling, inference and visualization.

## Availability and requirements

**Project name:** shinyBN.

**Project home page:** https://github.com/JiajinChen/shinyBN

**Operating system(s):**

For R users, any platform for which the R software is implemented;

For online users, any platform with compatible browser.

**Programming language:** R

**Other requirements:** Shiny

**License:** Apache License 2.0

**Any restrictions to use by non-academics:** None

## Supplementary information

**Additional file 1. **A zip archive containing the source codes and manual of *shinyBN*.

**Additional file 2. **The compatibility of the proposed *shinyBN* application. We tested the compatibility of *shinyBN* across three major operating systems and popular browsers.

**Additional file 3. **The inference result generated by *shinyBN*. (A) The settings of the evidence for some candidate gene loci; (B) The predicted probability of stroke displayed in a probabilistic table; (C) The predicted probability of stroke displayed in a bar plot.

**Additional file 4.** The ROC plot for the simulated validation set. This file contains the receiver operating characteristic curve for the simulated validation set of the stroke network.

**Additional file 5.** The DCA plot for the simulated validation set. This file contains the decision curve for the simulated validation set of the stroke network.

**Additional file 6.** An R script containing all the code to construct the stroke network using conditional probability tables provided in [1].

**Additional file 7. **A zip archive containing the testing files of *shinyBN*.

## Data Availability

The proposed *shinyBN* is deployed at https://jiajin.shinyapps.io/shinyBN/. Source codes and manual are freely available at https://github.com/JiajinChen/shinyBN and Additional file 1. The R scripts to construct the stroke network are included as Additional file 6. The testing files for users are included as Additional file 7.

## Abbreviations

API: Application programming interface
DCA: Decision curve analysis
HTML: HyperText Markup Language
ROC: Receiver operating characteristic
SCA: Sickle cell anemia
SMILE: Structural modeling, inference, and learning engine
SNP: Single nucleotide polymorphism
