# Supplementary figures and images for: shinyBN: an online application for interactive Bayesian network inference and visualization
Source: BMC Bioinformatics. 2019 Dec 16;20:711. doi: 10.1186/s12859-019-3309-0 (PMC6916222; doi:10.1186/s12859-019-3309-0)

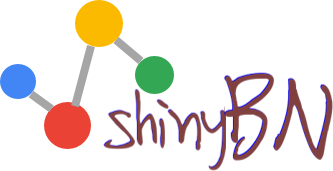

Supplement: Supplementary file 1 — Additional file 1. A zip archive containing the source codes and manual of shinyBN. [file 12859_2019_3309_MOESM1_ESM.zip › Additional file 1/shinyBN v1.0/www/logo2.png]

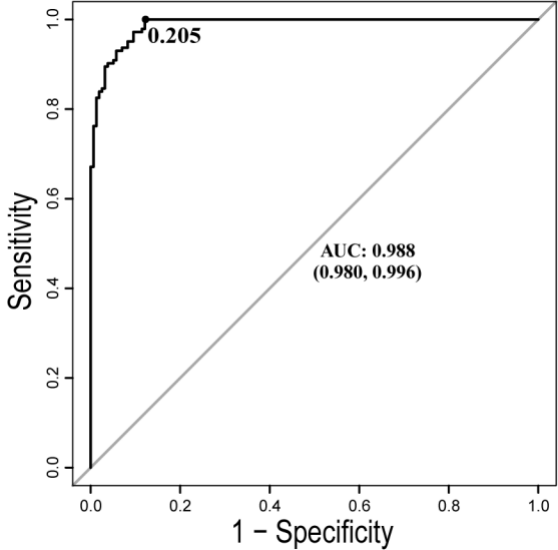

Supplement: Supplementary file 4 — Additional file 4. The ROC plot for the simulated validation set. This file contains the receiver operating characteristic curve for the simulated validation set of the stroke network. [file 12859_2019_3309_MOESM4_ESM.pdf]

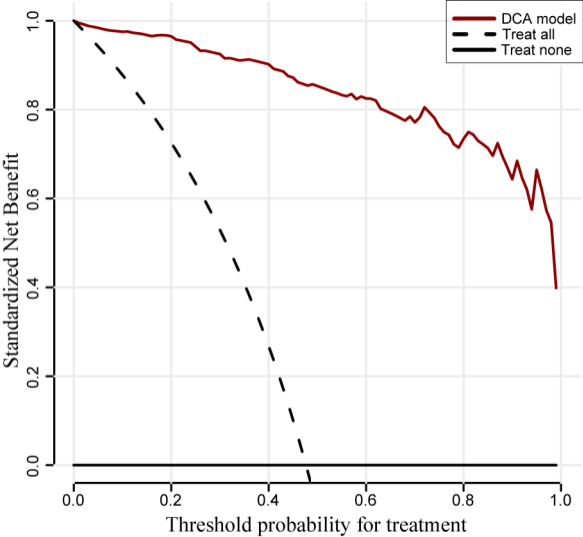

Supplement: Supplementary file 5 — Additional file 5. The DCA plot for the simulated validation set. This file contains the decision curve for the simulated validation set of the stroke network. [file 12859_2019_3309_MOESM5_ESM.pdf]
