# Supplementary material for: shinyBN: an online application for interactive Bayesian network inference and visualization
Source: BMC Bioinformatics. 2019 Dec 16;20:711. doi: 10.1186/s12859-019-3309-0 (PMC6916222; doi:10.1186/s12859-019-3309-0)
Supplement: Supplementary file 2 — Additional file 2. The compatibility of the proposed shinyBN application. We tested the compatibility of shinyBN across three major operating systems and popular browsers. [file 12859_2019_3309_MOESM2_ESM.docx]

**Additional file 2.** The compatibility of proposed *shinyBN*.

| OS | Version | R | Chrome | Firefox | Microsoft Edge | Safari |
| --- | --- | --- | --- | --- | --- | --- |
| Linux | Ubuntu | **√** | **√** | **√** | n/a | n/a |
| Mac OS | High Sierra | **√** | **√** | **√** | n/a | **√** |
| Windows | 10 | **√** | **√** | **√** | **√** | n/a |
