# Supplementary material for: shinyBN: an online application for interactive Bayesian network inference and visualization
Source: BMC Bioinformatics. 2019 Dec 16;20:711. doi: 10.1186/s12859-019-3309-0 (PMC6916222; doi:10.1186/s12859-019-3309-0)

(A)

Show 10 entries Search:

Evidence	Value
ANXA2.6	AA
BMP6.10	TT
BMP6.12	CC
SELP.14	CC
TGFBR3.10	CC
ERG.2	AA

Showing 1 to 6 of 6 entries Previous 1 Next

(B)

Show 10 entries

Variable	Level	Proability
STROKE	0	27.31%
STROKE	1	72.69%

Showing 1 to 2 of 2 entries Previous 1 Next

(C)

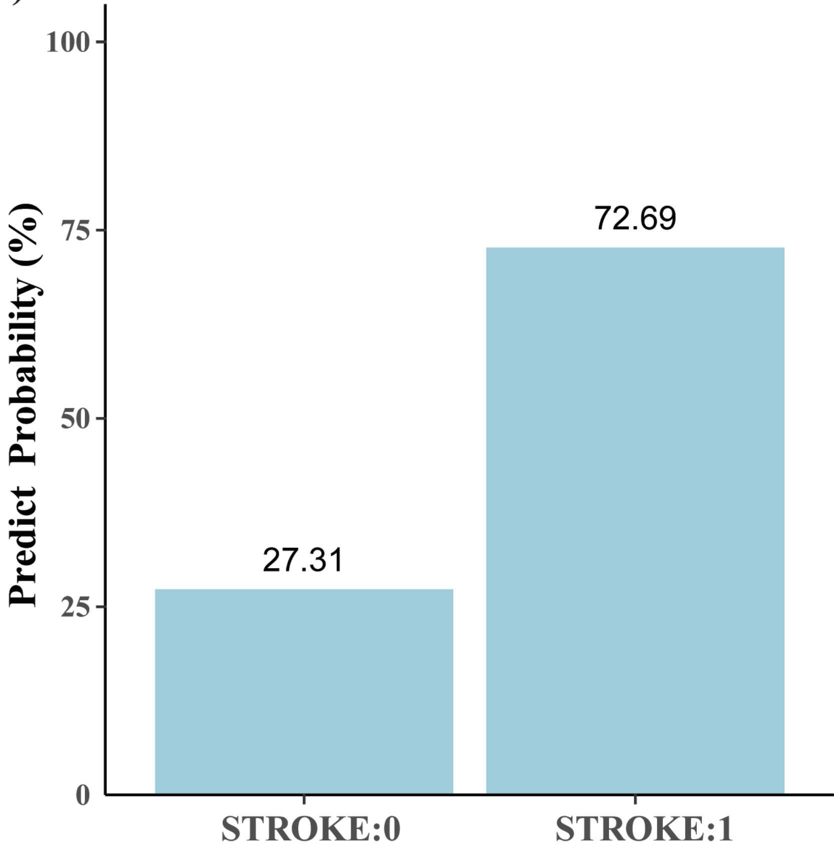

Supplement: Supplementary file 3 — Additional file 3. The inference result generated by shinyBN. (A) The settings of the evidence for some candidate gene loci; (B) The predicted probability of stroke displayed in a probabilistic table; (C) The predicted probability of stroke displayed in a bar plot. [file 12859_2019_3309_MOESM3_ESM.pdf]
